# Fine-Needle Aspiration Cytology for Parotid Tumors

**DOI:** 10.3390/life12111897

**Published:** 2022-11-15

**Authors:** Masataka Taniuchi, Tetsuya Terada, Ryo Kawata

**Affiliations:** Department of Otorhinolaryngology, Head and Neck Surgery, Osaka Medical and Pharmaceutical University, Takatsuki 569-8686, Japan

**Keywords:** fine-needle aspiration, cytology, parotid tumor

## Abstract

Fine-needle aspiration (FNA) cytology is widely used in clinical practice as a simple and minimally invasive test for parotid tumors that allows for preoperative estimation of benignancy and malignancy, histological type, and malignancy grade and can be performed on an outpatient basis. In recent years, cell blocks prepared with core needle biopsy (CNB) and liquid-based cytology (LBC) have increased the reliability of immunostaining and molecular biological testing, leading to improved diagnostic accuracy. In 2018, the Milan System for Reporting Salivary Gland Cytology was introduced, but it does not include malignancy grade or histological type, so we proposed the Osaka Medical College classification as a more clinically based cell classification that includes both types of information, and we have reported on its usefulness. This review gives an overview of the history and use of FNA and describes CNB and LBC and the two classification systems.

## 1. Introduction

Fine-needle aspiration (FNA) cytology with a small-bore needle is widely used in clinical practice as a simple and minimally invasive test for parotid tumors that allows for the preoperative estimation of benignancy and malignancy, histological type, and malignancy grade before surgery and can be performed on an outpatient basis [1,2,3]. In addition to differentiating between neoplastic and non-neoplastic lesions, FNA cytology can often be used to diagnose histological type in neoplastic lesions, such as benign tumors such as pleomorphic adenoma and Warthin’s tumor [4,5,6,7,8,9,10,11,12,13], or to determine the malignancy grade in the case of malignant tumors.

The use of FNA cytology for preoperative diagnosis requires that It not only enables differentiation between benign and malignant tumors but also helps determine the best course of treatment, especially surgical treatment. Detailed preoperative information is important because histological types such as pleomorphic adenoma and Warthin’s tumor, which account for the majority of benign tumors, require different treatments [14,15]. In the case of pleomorphic adenoma, for example, surgical resection is indicated, and the operative procedure needs to avoid enucleation. In the case of Warthin’s tumor, the possibility of malignant transformation can be ruled out, and as long as there is no problem with cosmetic aspects, a wait-and-see approach may be an option [16,17]. On the other hand, in malignant neoplasms, it is important to diagnose the grade of malignancy to determine subsequent treatment strategies. The prognosis differs significantly between low- or medium-grade and high-grade malignant cancers, and operative strategies, such as facial nerve treatment and indications for cervical lymph node dissection, vary accordingly [18,19,20,21,22,23,24,25,26].

The sensitivity and specificity of parotid gland FNA cytology depend on the operator’s technique, the quality of the specimen, the pathologist’s experience, and the presence and absence of cystic components [4,5,6,7,8,9,10,11,12,13,14,15,27,28]. However, the most relevant aspect for differential diagnosis is the wide variety of histological types of parotid tumors: There are 23 types of malignant tumors and 11 types of benign tumors [29].

In view of the above, in 2018, an international group of pathologists proposed a standardized reporting system for the stratification of malignancy grades, the Milan System for Reporting Salivary Gland Cytopathology [30]. However, the Milan System does not consider aspects related to the histological type or malignancy grade, so at our hospital, we developed a new classification, referred to as the Osaka Medical College (OMC) classification, and examined its adequacy and usefulness [31].

In this review, we describe the history of FNA; features of parotid cytology; specimen collection and processing procedures, including FNA, core needle biopsy (CNB), and liquid-based cytology (LBC); frozen section biopsy (FSB); ancillary diagnosis; and the advantages and disadvantages of the Milan and OMC classification systems.

## 2. History of Fine Needle Aspiration

According to various publications, FNA was first reported by Kun in 1847 [32], and in 1904, Grie et al. used lymph node FNA to diagnose cases of sleeping sickness caused by parasites (Trypanosomiasis) [33]. Martin and Ellis et al. used cytology for head and neck diseases in 1930 [34], and since then, FNA has been used for a variety of organs, including lymph nodes, bone, lungs, prostate, and mammary glands. However, until the 1950s, FNA was mainly performed with a large-bore needle (14–16G) and did not gain widespread use because of complications such as bleeding and neoplasm seeding associated with cell and tissue sampling. In the 1950s, Franzen et al. performed FNA with a small-bore needle (22–23G) [35], and in the 1970s, Frable and Frable introduced FNA for head and neck tumors [36]. Since then, FNA has been widely used in clinical practice.

In terms of cytology diagnosis, in 1928, Babeş and Papanicolaou separately reported that smear samples of cells collected from the vaginal portion of the uterus could be used for diagnosis [37,38]. Then, in 1943, Traut and Papanicolaou proposed the Papanicolaou classification, which divided the properties of cells observed in smear samples of the vaginal portion of the uterus into five classes (Roman numerals I–V) according to the “cell malignancy grade”; the authors demonstrated the usefulness of this classification in screening for cervical cancer [39]. The Papanicolaou classification focuses on the evaluation of cell malignancy grade, which is easy to understand, practical, and therefore useful; consequently, its application has gradually been extended to the cytologic diagnosis of other organs. However, the Papanicolaou classification is more of a screening test than a diagnostic tool and is associated with problems such as discrepancies between cytology diagnosis and histology diagnosis. Therefore, in 1988, the Bethesda system for cervical cytology was established and led to the development of unique assessment classifications for each organ, including the Bethesda system for thyroid cytology in 2010 [40], followed by the establishment of the Milan System [30]. There are plans to publish the second version of the Milan System in 2023, and various authors have reported a variety of proposed revisions and modifications [41,42,43,44].

## 3. Features of Parotid Gland Cytology

Parotid tumors comprise 23 histological types of malignant tumors and 11 types of benign tumors [29]. For this reason, FNA cytology is useful for differential diagnosis, although it is also associated with diagnostic difficulties. Among the benign tumors, two histological types—pleomorphic adenoma and Warthin’s tumor—account for roughly 90% of all cases, and FNA cytology allows for their histological differentiation [14,15]. On the other hand, among malignant tumors, a large number of histological types and the presence of histological subtypes often make the diagnosis of histological type with cytology quite difficult.

In regard to the cytodiagnosis of salivary gland tumors, the first question is whether the tumor is neoplastic or non-neoplastic; determining whether a tumor is a neoplasm is often difficult in the case of cystic lesions or lesions with lymphocytic infiltration. Salivary gland epithelial cells include lumen-forming cells (acinar cells and ductal epithelial cells) and non–lumen-forming cells (myoepithelial cells and basal cells). In the case of salivary gland tumors, the first step of diagnosis is to differentiate between myoepithelial/basal cell-related tumors, as represented by polymorphic adenoma and basal cell adenoma, and non-myoepithelial/basal cell-related tumors, as represented by Warthin’s tumor. When used in combination with FNA cytology, Giemsa staining helps identify myoepithelial/basal cell-related tumors. Dyskaryosis is an important finding for differentiating between benign and malignant tumors, and evaluation methods used to determine the malignancy grade differ depending on histological types, such as mucoepidermoid carcinoma and adenoid cystic carcinoma [45,46,47,48].

## 4. Specimen Collection and Processing Procedures

Because FNA cytology has some disadvantages, CNB and LBC were recently introduced as new sample collection and processing procedures, respectively. All three procedures are described below, and the advantages and disadvantages of each procedure are summarized in Table 1.

### 4.1. Fine Needle Aspiration Cytology

In parotid gland FNA cytology, appropriate specimen collection, and processing are important. In order to obtain samples, a 22 to 23-gauge needle is attached to a 10-cc syringe, and a puncture is made into the mass under ultrasound guidance. Then, the needle is moved back and forth to make sure that it reaches the deepest part of the lesion, and aspiration is performed. For aspiration, pressure is applied by pulling the syringe plunger. Once the aspiration is complete, the negative pressure is released (by returning the syringe plunger to the original position), the needle is removed, and compression is applied to the puncture site. It is advisable to prepare the sample immediately after aspiration; usually, immediately after smearing the cells onto a sliding glass, the sample is fixed with 95% ethanol and then stained with Papanicolaou stain. In addition, the smeared cells are dried, and after methanol fixation, Giemsa staining is performed. In order to improve the rate of correct diagnosis; re-puncture is performed if the specimen is bloody to ensure that the sample includes tumor cells. If possible, rapid onsite cytodiagnosis should be performed to evaluate the adequacy of the specimen [49].

### 4.2. Core Needle Biopsy

CNB is a biopsy method in which tissue is removed with a thicker needle (8–20 G) than that used in FNA. Compared with FNA, CNB allows for the collection of larger tissue samples with preserved tissue structure [50], enabling general tissue and immunohistochemical staining and, thus, a more reliable histological diagnosis than is possible with standard cytology. Although CNB is generally considered safe, the use of a thicker needle can increase the risk of complications, such as tumor seeding, facial nerve damage, and hematoma [51,52,53,54,55]. However, according to a review by Shah et al., seeding after CNB in the head and neck region is rare, and the risk of local recurrence is roughly 0.0011% with CNB versus 0.00012% with FNA, indicating that the risk of seeding is not significantly different between FNA and CNB [56]. In a review by Schmidt et al., the sensitivity, specificity, and rate of inadequate specimen collection of CNB were reported to be 92%, 100%, and 1.2%, respectively, demonstrating that the sensitivity is higher and the rate of inadequate specimen collection is lower than with FNA [57]. Similarly, Cengiz et al. also noted that CNB has a higher diagnosis rate than FNA cytology [58]; the group pointed out the usefulness of CNB but admitted that there is room for argument regarding its risks, such as tumor seeding.

### 4.3. Liquid-Based Cytology

In LBC, sampled cells are stored as a cell suspension solution, and cytology specimens are prepared later with a special instrument. The specific advantages of this method are that the background contamination with blood can be cleaned, artifacts from air-drying are reduced, quick and proper fixation can be performed, samples can be preserved for a longer time, and specimens can be created with uniform density because the cells are smeared onto a limited circular area of the slide. However, the procedure also has some disadvantages in that cytomorphological, and sample background findings differ from those of conventional specimens because of the decreased cell cluster size and reduced extracellular particles [59,60,61]. Nevertheless, in addition to being used for the preparation of cytology specimens, the remaining samples can be used for tests such as immunocytochemistry and molecular chemical analysis [62,63]. Given these advantages, LBC is considered to enable a more accurate diagnosis than is possible with conventional FNA cytology. Kumar et al. examined the use of LBC in parotid tumors and found that, although LBC had comparable specificity and sensitivity to FNA cytology, it was better for observing nuclear details and background [64]. Another advantage of LBC is that cell blocks can be prepared and used for immunostaining [65].

## 5. Ancillary Diagnosis in Salivary Gland Cytology

Diagnosing salivary gland tumors by conventional cytology alone is difficult in some cases, so various ancillary diagnostic procedures are used. These procedures include staining methods, fluorescent in situ hybridization (FISH), and reverse transcription polymerase chain reaction (RT-PCR) [66,67,68,69,70,71,72,73,74,75].

Most immunostaining and molecular biological tests can be applied to a variety of cytology specimens, such as alcohol-fixed, dry-fixed, and LBC specimens, but using formalin-fixed, paraffin-embedded cell block sections is considered to be more reliable [67,68,70]. Immunostaining is used to support differential diagnosis in cases with an unclear diagnosis. Because each tumor can have a wide range of differential diagnoses (e.g., basaloid tumor, eosinophilic tumor, clear cell tumor), it is desirable to perform immunostaining with multiple antibody panels [69]. Recently, specific, highly reproducible gene translocations were found in salivary gland tumors [66,67,68,76,77,78,79,80]. Fusion oncogenes and oncoproteins resulting from gene translocations are useful diagnostic markers and can be detected by FISH and RT-PCR. Previously reported gene translocations and fusion oncogenes include the CRTC1/3-MAML2 fusion gene in mucoepidermoid carcinoma and the MYB-NFIB fusion gene in adenoid cystic carcinoma [81,82,83]. Although these genetic abnormalities are highly specific and can be considered as powerful diagnostic markers, detection of genetic abnormalities, in general, is not always reliable, so certain types of salivary gland tumors should not be excluded only on the basis of genetic testing. In the future, the detection of genetic abnormalities may become useful not only as an indicator for tissue diagnosis and prognosis but also as a target for gene therapy [76,79].

## 6. Frozen Section Biopsy

In parotid surgery, FSB has the following three general roles: to clarify the diagnosis, to check the resection stump, and to determine the presence or absence of nerve and cervical lymph node infiltration [84]. As FNA cytology became more commonly used for preoperative diagnosis in parotid surgery, opportunities to perform FSB became scarce, and its role shifted from being primarily a tool to differentiate between benign and malignant tumors and diagnose histological type to a method for assessing margins [85]. Although performing FSB may improve preoperative diagnosis in parotid surgery, it remains unclear whether FSB should always be used or whether its use should be limited to specific situations. Some authors suggest that FSB is more reliable than FNA cytology and that it should be used on a routine basis to ensure the best patient management [86]; on the other hand, others advocate using FSB only under certain circumstances, such as in cases with uncertain preoperative FNA cytology [87,88,89,90].

In their study on the usefulness of FSB for preoperative diagnosis based on FNA cytology, Choy et al. reported that the risk of malignancy (ROM) increased to as high as 100% when the result of FSB was “malignant” in cases with a diagnosis of atypia of undetermined significance (AUS), salivary gland neoplasm of uncertain malignant potential (SUMP), or suspicious for malignancy based on FNA cytology [87]. Pastorello et al. also found that the use of FSB in specimens diagnosed as non-diagnostic or SUMP by FNA cytology can increase specificity and positive predictive value [88]. Thus, FSB can potentially help in cases where FNA cytology does not lead to a benign/malignant diagnosis.

If FNA cytology does reveal a benign diagnosis, such as polymorphic adenoma or Warthin’s tumor, the effectiveness of FSB is limited because the risk of malignancy is very low; hence, in such cases, there is likely to be little need for FSB [89]. Disease stage and histological grade are important prognostic factors for parotid cancer, and the histological type and grade of tumors need to be accurately determined before surgery to allow surgeons to decide on the necessary extent of local resection, whether the facial nerve can be preserved or not, and whether neck dissection is necessary. Nishikawa et al. reported that compared with FNA cytology alone, additional FSB increases the accuracy of the histological grade [91]. If malignancy is suspected on FNA cytology, performing FSB concomitantly might allow the appropriate range of resection in parotid cancer surgery to be determined [90].

## 7. Classification Systems

### 7.1. Milan System

As mentioned above, the Milan System was introduced in 2018 as a system for reporting the classification of salivary gland cytology [30]. It was created by a group of pathologists and divides tumors into six groups, including a non-diagnostic category, whereby group IV comprises two subgroups. The aim of the group was to develop a diagnostic reporting system that would allow for the stratification of malignancy grades. Hence, ROM and clinical response are indicated for each category [5,6,7,9,10,92]. The ingenuity of this system lies in the classification of cases for which benign and malignant differentiation is difficult; such cases are divided into AUS, SUMP, and suspicious for malignancy. This section describes the classes of the Milan System and the appropriate clinical measures for each class. The classes of the Milan System are described below:

#### 7.1.1. I. Non-diagnostic (ROM 25%)

Specimens that are insufficient in terms of quantity and quality and do not provide useful information for making a diagnosis. Although there is no set standard for “diagnostic” specimens, the Bethesda System for Reporting Thyroid Cytopathology uses a cutoff of at least 60 cells derived from the lesion as an index [30,93]. Specimens diagnosed as non-diagnostic include those containing no or almost no cellular components, those for which evaluation of cellular components is difficult due to dryness or blood contamination, and those with non-mucinous cyst fluid that does not include epithelial components. However, even when the number of cells is below the cutoff value, if cellular atypia is observed, mucinous cysts that do not include epithelial components are diagnosed as AUS, as described below. Ideally, 10% or fewer of specimens should be classified as non-diagnostic.

**Clinical measures:** Repeat FNA cytology should be performed on specimens.

#### 7.1.2. II. Non-neoplastic (ROM 10%)

Commonly observed non-neoplastic lesions include inflammatory diseases such as acute salivary adenitis and chronic salivary adenitis, including granulomatous lesions [94,95,96,97]. This category includes bacterial infections in acute salivary adenitis; diseases that cause obstruction of the salivary gland duct, such as sialolithiasis, and IgG4-related diseases in chronic salivary adenitis; and mucocele and sarcoidosis in granulomatous inflammation [1,6,95,96,97]. To diagnose a specimen as non-neoplastic, physicians must perform a comprehensive assessment and always refer to clinical and imaging information.

**Clinical measures:** Physical and imaging findings are re-examined, and the patient is followed up. If imaging shows changes or a patient’s symptoms worsen, repeat FNA cytology should be performed.

#### 7.1.3. III. AUS (ROM 20%)

This category is used for specimens with qualitatively or quantitatively insufficient cytological findings for diagnosing whether they are non-neoplastic or neoplastic. Specific examples include cellular atypia for which the possibility of neoplastic disease cannot be ruled out; cases in which changes in the squamous epithelium or eosinophilic changes are observed; neoplastic disease is suspected, but the number of cells is small; epithelial components are observed, but the presence of blood and other substances makes evaluation difficult; mucinous cystic lesions with few epithelial components; and lymphoid lesions that cannot be definitively diagnosed as lymphoproliferative disease. These specimens may have a low number of cells but show cellular atypia and thus cannot be categorized as non-diagnostic. As with non-diagnostic specimens (I.), ideally, fewer than 10% of cases should be identified as AUS.

**Clinical measures:** Physicians should also consider other types of clinical information, such as imaging and physical findings, and decide whether to perform repeat FNA, biopsy, or surgical resection.

#### 7.1.4. IV. Neoplasm

As described above, FNA cytology enables differentiation between both non-neoplastic and neoplastic diseases and benign and malignant diseases with high specificity. In particular, polymorphic adenoma and Warthin’s tumor can be diagnosed with high specificity. However, for other histological types, tissue typing can be difficult without the use of ancillary diagnostic procedures or other approaches. The difficulty with tissue typing is probably a result of the diversity of histological types of salivary gland tumors and the variety of types observed even within the same tumor (although cytological findings of salivary gland tumors of different histological types do share similarities). Therefore, it is difficult to differentiate between benign and low-grade malignant tumors, so two subcategories of neoplasms were established:

##### IVA. Neoplasm—Benign (ROM ≤ 5%)

Clearly benign tumors, pleomorphic adenoma and Warthin’s tumor account for the majority of cases.

##### IVB. Neoplasm—SUMP (ROM 35%)

The specimen is clearly a neoplasm as judged from the cellular morphological features, but it is difficult to determine whether it is benign or malignant. This category includes hypercellular basaloid and hypercellular eosinophilic cell tumors for which a benign/malignant diagnosis is difficult to make by cytology and for which vascular and nerve infiltration needs to be diagnosed histologically. Such tumors include basal cell adenoma/basal cell adenocarcinoma, myoepithelial tumors, myoepithelial carcinoma, clear cell tumors, and low-grade malignant tumors with limited atypical features.

**Clinical measures**: If the specimen is diagnosed as Neoplasm—Benign, diagnostic imaging is performed, and the lesion is removed surgically after its location and size have been determined. In the case of parotid tumors, an appropriate operative approach (e.g., extracapsular dissection of tumors, lobectomy) is chosen after explaining to patients the risk of facial nerve paralysis, depending on the location of the lesion (e.g., whether it is located in the superficial or deep lobe). In addition, if Warthin’s tumor is strongly suspected after performing gadolinium contrast-enhanced magnetic resonance imaging (MRI) or another suitable imaging procedure, observation may also be chosen as the management approach. The measures to take are the same if the specimen is diagnosed as Neoplasm—SUMP, but the rapid intraoperative diagnosis should be performed because the likelihood of low-grade cancer is high. Depending on the result, an additional excision, such as neck dissection, may be indicated.

#### 7.1.5. V. Suspicious for Malignancy (ROM 60%)

Although the sample does not have all the findings of malignancy, the findings suggest malignancy as a whole. Specimens in this category are mostly those with blood contamination or insufficient cell sampling, even though the specimens contain highly atypical cells. Other examples include specimens with minor cytological findings specific to malignant lesions, specimens with mixed malignant and benign findings, and specimens for which malignant lymphoma is suspected but difficult to determine. In some cases, these specimens can be diagnosed as VI. Malignant by performing ancillary diagnostic tests.

**Clinical measures:** In order to determine whether or not a tumor is malignant, repeat FNA, needle biopsy, incision biopsy, surgical resection, or other suitable tests are performed; physicians must consider whether an additional specimen should be obtained.

If surgical resection is performed after images, such as from MRI and computed tomography (CT), have been closely examined and the range of lesions, including metastatic lesions and disease stage, have been further evaluated, a rapid intraoperative pathological diagnosis should be obtained to evaluate whether neck dissection is indicated, and postoperative radiotherapy should be performed. If surgical resection is not indicated, the diagnosis should be confirmed by repeat FNA cytology, biopsy, and, if possible, ancillary diagnostic tests, and the further course of treatment should be decided.

#### 7.1.6. VI. Malignant (ROM 90%)

The specimen can be determined as malignant based on cytological findings alone or in conjunction with an ancillary diagnosis. If surgery is indicated, the histological type and malignancy grade should also be described, if possible, because they are closely related to the extent of resection and the decisions on whether or not neck dissection should be performed and, in the case of parotid tumors, whether or not the facial nerve should be preserved.

**Clinical measures:** Factors that determine the prognosis of parotid cancer include T classification, histological malignancy grade, facial nerve infiltration, and old age; among these, advanced T and histological malignancy grades are known to be the most important factors [18,19]. The extent of local progression should be determined by close examination of CT, MRI, and positron emission tomography-CT (PET-CT) images. As for the malignancy grade, a diagnosis should be made by FNA to the extent possible, and surgical plans should be developed accordingly. In many cases, it is difficult to establish a diagnosis, including the histological type, by FNA; thus, rapid intraoperative cytology should also be used to diagnose the histological type and malignancy grade. In terms of local operative procedures, partial lobectomy is indicated if the patient has a relatively small low- to the medium-grade tumor [98]. In the case of advanced T-stage or highly malignant cancer, subtotal, total, and extended total parotidectomy are indicated [99]. With respect to the facial nerve, if nerve infiltration is observed, nerve excision is performed regardless of the grade of malignancy. With regard to neck dissection, patients with lymph node metastasis are known to have a poor prognosis [20,21]; however, the usefulness of therapeutic neck dissection in such patients and the best treatment strategies in patients without clinical lymph node metastasis have not been established. A number of reports have described total neck dissection in patients with positive lymph node metastasis [22,23], and some reports suggest that preventive neck dissection should be performed for high-grade and advanced T cancers [24,25,26]. In cases without an indication for surgery, repeat FNA cytology, incision biopsy, and, if possible, ancillary diagnostic tests should be used to establish the diagnosis, and treatment plans such as radiotherapy and chemotherapy should be developed accordingly.

### 7.2. Osaka Medical College Classification

The Milan System does not include malignancy grade or histological type, but such information is useful preoperatively for planning the course of treatment for salivary gland tumors. Therefore, our group proposed the OMC classification as a cytology classification that better matches clinical needs by incorporating the histological type and malignancy grade. The OMC classification has 11 categories, and the main difference from the Milan System is that tumors classified as IVA Neoplasm—Benign are further classified into histology confirmed (4-1) or histology unconfirmed (4-2). Moreover, those classified as VI. Malignant is divided into three categories: malignant (grade/histology unconfirmed) (6-2), malignant (grade confirmed) (6-3), and malignant (grade/histology confirmed) (6-4) (Table 2).

We verified the adequacy and validity of this classification system by examining 1175 patients with parotid tumors (benign tumors, n = 981; malignant tumors, n = 194; among the malignant tumors, 113 were low-/medium-grade malignancy and 81 were high-grade malignancy) who underwent surgery after preoperative FNA cytology at our department and in whom the OMC classification was applied. The risk of malignancy (ROM) was also calculated for each FNA diagnosis (Table 3). In the OMC classification, the ROM was comparable to the result of the Milan system. Accordingly, it is considered that this new classification system was appropriate in terms of differentiating between benign and malignancy. The FNAC diagnosis (OMC classification) was evaluated based on the final pathological diagnosis (Table 4). For 51 patients with mucoepidermoid carcinoma (low/intermediate-grade: 26 patients, high-grade: 25 patients), 27 patients (52.9%) were diagnosed as “malignant” (Category 6). Grade of malignancy (Category 6-3 + 6-4) was determined in 19.2% of low/intermediate-grade and 60.0% of high-grade. Other histology types are shown in Table 4. The FNAC diagnosis (OMC classification) was reviewed for patients whose final pathological diagnosis was a benign tumor, including pleomorphic adenoma (605 patients), Warthin tumor (225 patients), or basal cell adenoma (47 patients). The percentage of patients in whom benign histology was diagnosed by OMC classification (Category 4-1) was 85.1%, 73.8%, and 44.9%, respectively. Of the 981 cases with benign tumors as diagnosed by FNA cytology, 717 (73%) were diagnosed as OMC category 4-1, i.e., with confirmed histology. Moreover, when the 717 cases were compared relative to the final pathology, 93% were diagnosed correctly. Therefore, we concluded that the histological type of benign tumors can be determined by FNA cytology and that it is reasonable to include the histological type in the classification of cytology. However, malignant tumors diagnosed as category 6-4, i.e., with confirmed grade and histology, accounted for only 31% of all malignant tumors, and of these, 62% were diagnosed correctly. With regard to the malignancy grade, of the 194 cases with malignant tumors as diagnosed by FNA cytology, 82 (44%) were diagnosed, including the malignancy grade (categories 6-3 and 6-4). Moreover, when the 82 cases were compared relative to the final pathology, 88% were diagnosed correctly. Therefore, we concluded that FNA cytology can be used to determine the malignancy grade of malignant tumors and that it is reasonable to include the malignancy grade in the classification of cytology.

When taken together, FNA cytology for salivary gland tumors can be used to diagnose the histological type and malignancy grade, and because it has a good rate of correct diagnosis, it is reasonable to include histological type in the salivary gland cytology classification. Therefore, the OMC classification is considered clinically useful [31].

## 8. Conclusions

We have described the development of cytopathology for salivary gland tumors. In recent years, the accuracy of cytopathological diagnosis has improved with the development of specimen collection and system of diagnosis. The Milan system, which was a classification of cytopathology for salivary gland tumors, showed the risk of malignancy in each FNA category. We recently proposed a new classification (OMC classification) which included histological type and grade of malignancy in the classification category. We have concluded that FNA for the salivary gland tumors contributed to the diagnosis of not only benign/malignant but also histological type and grade of malignancy, and this information will help us to determine the treatment strategy.

## Figures and Tables

**Table 1:** Advantages and disadvantages of fine needle aspiration, core needle biopsy, and liquid-based cytology.

Procedure	Advantages	Disadvantages
Fine needle aspiration	Rapid, simple, and minimally invasive Cost-effective	The rate of inadequate specimen collection is higher
Core needle biopsy	High sensitivity and specificity Immunostaining and molecular biological tests can be performed	Invasive, possible risk of tumor seeding Requires skill
Liquid-based cytology	Cell specimens can be preserved Better observation of nuclear details and background Immunostaining and molecular biological tests can be performed	Costly Changes may occur in cytomorphological findings and sample background findings

**Table 2:** Comparison of the Milan System and Osaka Medical College classification.

Milan System		Osaka Medical College Classification
I. Non-diagnostic	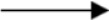	1-1 Inadequate
	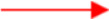	1-2 Cyst contents
II. Non-neoplastic	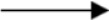	2 Non-neoplastic
III. AUS	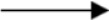	3 AUS
IV. Neoplasm	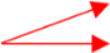	4-1 Benign tumor (histology confirmed)
IVA. Neoplasm—benign		4-2 Benign tumor (histology unconfirmed)
IVB. Neoplasm—SUMP	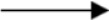	5 SUMP
V. Suspicious for malignancy		6-1 Suspicious for malignancy
VI. Malignant	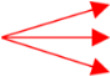	6-2 Malignant (grade/histology unconfirmed)
		6-3 Malignant (grade confirmed)
		6-4 Malignant (grade/histology confirmed)

AUS, atypia of undetermined significance; SUMP, salivary gland neoplasm of unknown malignant potential.

**Table 3:** Cytopathological results for the Osaka Medical College (OMC) system for salivary gland tumors.

	1-1	1-2	2	3	4-1	4-2	5	6-1	6-2	6-3	6-4
Malignant (n = 194) (113/81)	26 (18/8)	2 (2/0)	5 (4/1)	0	12 (9/3)	13 (13/0)	23 (15/8)	13 (10/3)	18 (7/11)	21 (6/15)	61 (29/3)
Benign (n = 981)	133	20	27	8	717	38	26	4	3	1	4
Total (%)	13.5	1.9	2.7	0.7	62.0	4.3	4.2	1.4	1.8	1.9	5.5
ROM (%)	16.4	9.1	15.6	0.0	1.6	25.5	46.9	76.5	85.7	95.5	93.8

The number in parentheses indicates each case by histological grade (low- or intermediate-/high-grade). ROM, risk of malignancy.

**Table 4:** Cytopathological results for the Osaka Medical College (OMC) system for each final pathology.

	1-1	1-2	2	3	4-1	4-2	5	6-1	6-2	6-3	6-4	Total
Malignant												
MEC	8 (6/2)	1 (1/0)	3 (2/1)	0	2 (1/1)	3 (3/0)	7 (4/3)	4 (3/1)	3 (1/2)	3 (1/2)	17 (4/13)	51
Ca ex pleo	2 (1/1)				4 (2/2)	3 (3/0)	3 (0/3)	1 (0/1)	6 (2/4)	4 (1/3)	6 (2/4)	29
AdCC	3 (2/1)				1 (1/0)	2 (2/0)	3 (3/0)	1 (1/0)	3 (0/3)		9 (8/1)	22
SDC	1						1		1	8	7	18
Benign												
Pleo	40	3	7	3	515	16	12	2	3	1	3	605
Warthin	39	2	10	3	166	6	3	1				225
BCA	11				21	11	3				1	47

The number in parentheses indicates each case by histological grade (low- or intermediate-/high-grade). AdCC, adenoid cystic carcinoma; BCA, basal cell adenoma; Ca ex pleo, carcinoma ex pleomorphic adenoma; MEC, mucoepidermoid carcinoma; Pleo, pleomorphic adenoma; SDC, salivary duct carcinoma; Warthin, Warthin tumor.

## Data Availability

Not applicable.
